# The Major Qualitative Characteristics of Olive (*Olea europaea* L.) Cultivated in Southwest China

**DOI:** 10.3389/fpls.2017.00559

**Published:** 2017-05-19

**Authors:** Zizhang Cheng, Mingming Zhan, Zeshen Yang, Kristina Zumstein, Huaping Chen, Qianming Huang

**Affiliations:** ^1^Department of Biochemistry, College of Science, Sichuan Agricultural UniversityYaan, China; ^2^Sichuan Liangshan New Technology Development Co., Ltd.Xichang, China; ^3^Department of Plant Biology, University of California, DavisDavis, CA, USA

**Keywords:** olive fruit, oil content, sugar, phenolics, flavonoids, fatty acid composition

## Abstract

Olive trees, originated from Mediterranean, have been cultivated in China for decades and show great adaption to local environment. However, research on this topic is limited. In this study, the major qualitative characteristics and changes of olive grown in southwest China were investigated. The results showed that oil accumulated during fruit development and reached its maximum value when fruit had fully ripened. Phenolic and flavonoid contents increase rapidly in the early growth stage (0–90 DAFB) and then begin to decrease as fruit ripens. Compared with olive from the Mediterranean, olive from China has special characteristics: higher moisture content in the fruit combined with lower percentages of unsaturated fatty acids and oil content. This is due to southwest China's climate which is wetter and cooler compared to the Mediterranean. Our study suggests that southwest China's higher annual rainfall might contribute to higher fruit moisture content while its low temperatures would be conducive to higher unsaturated fatty acid levels in the fruit.

## Introduction

The olive (*Olea europaea* L.) is a famous edible oil tree crop worldwide and has great commercial value due to its peculiar nutritional benefits (Conde et al., 2008). Due to the well-balanced oil composition (highly enriched in monounsaturated fatty acid) and rich minor components (such as polyphenols and phytosterols) in the fruits, olive trees are unique among oil plants (Sánchez and Harwood, 2002). In order to understand the specific properties of olive oil and fruit, many studies have investigated olive fruits in the past few years (Nergiz and Engez, 2000; Brahmi et al., 2013; Cecchi et al., 2013).

The major qualitative characteristics of olive fruits include fruit weight, oil content, phenolics profile, and fatty acid composition, etc. In plants, the biosynthesis of these biochemical components are strongly influenced by genetic factors. Different varieties usually show different qualitative characteristics. For example, *O. europaea* cv. *Koroneiki* has a high phenolic content while cv. *Frantoio* has a high oil content (Gómez-Rico et al., 2008; Alu'datt et al., 2013; Franco et al., 2014; Sousa et al., 2014). However, in recent years, many studies have shown that environmental factors (such as temperature and moisture) also play an important role in determining olive qualitative characteristics (Ranalli et al., 1999; Vinha et al., 2005; Temime et al., 2006; Bakhouche et al., 2013). Climate (especially rainfall and temperature) influences the olive oil constituents significantly (Ranalli et al., 1999; Ocakoglu et al., 2009). The fruitiness is negatively affected by maximum air temperature. Too much rainfall in the fruiting season decreases the phenolic content but only slightly influences oil content and fatty acid composition (Ranalli et al., 1999; Patumi et al., 2002). Several studies has shown that geographical factors such as altitude strongly affect the qualitative characteristics (Arslan et al., 2013; Bajoub et al., 2015). Olives planted in high-altitude locations are rich in monounsaturated fatty acids (MUFA), while olives planted in low-altitude locations are rich in saturated fatty acids (SFA) (Nergiz and Ergönül, 2009). In addition, development stage also has a great effect on qualitative characteristics in fruits (Trentacoste et al., 2010; Gómez-González et al., 2011). During fruit ripening, oil content and fruit weight increase rapidly, and the accumulation patterns follow an “S” curve. However, the accumulation curves of other components, such as phenolics, reach the peaks when fruits are mature. Thus, an appropriate harvesting time is essential for olives.

Although many studies have investigated the biological characteristics of olive fruits in recent years, these studies mostly focus on olives planted in several countries in the Mediterranean region. China began to plant olives in the 1960's, and currently, olives are mainly planted in Southwest China. After being cultivated for half a century, olives have adapted to the local environment and have given rise to new varieties such as *Ezhi*. However, reports on these new cultivars are rare. Hence, we performed this study to reveal the qualitative characteristics of these olive fruits. Our work also provide supportive information for olive cultivation in other regions.

## Materials and methods

### Plant material

Olive fruits were collected from commercial olive orchards located in Xichang, Sichuan (34°87′N, 15°07′E, altitude of 1500~2000 m), southwest China, the main olive planting region in China. Ten olive cultivars that are widely planted in Southwest China were used for the analyses (Table 1). In orchards, olive trees (15 years old) having a vase shape with 5x8 distances were cultivated. Pruning was done every 1–2 years and irrigation was performed as 1–1.4 ET_0_ per watering in drought season (from November to June).

**Table 1:** **The origin of olive cultivars that planted in China**.

**Varieties**	**Origin place**	**Varieties**	**Origin place**
*Yuntai*	China	*Arbequina*	Spain
*Frantoio*	Italy	*Koroneiki*	Greece
*Ezhi-8*	China	*Coratina*	Italy
*Picual*	Spain	*Barnea*	Israel
*Leccino*	Italy	*Ottobratica*	Italy

The fruits were collected in two harvest years (2014 and 2015). In each year, 3 trees in total from three olive areas with uniform characteristics were chosen for sampling. About 300 fruits were collected in each olive growth zone. Olive fruits without damage were collected by hand from olive tree every 30 days after flowers full bloom (DAFB, from May to November). During the olive harvest time (150–180 DAFB), undamaged fruits of each variety were collected separately once every 10 days. And then for each variety, we mixed fruits collected every 10 days. The fresh fruits were washed with distilled water. Olive fruits from each variety were weighed at each sampling time. Then the fruits were immediately homogenized with a laboratory crusher at 4°C and lyophilized until they reached a constant weight. The moisture content was calculated, and the lyophilized paste was stored at −20°C. The fruits of each year were measured separately. The data was pooled together for statistical analysis and evaluation.

### Oil content

The oil content was determined by extracting the lyophilized paste with petroleum ether using a Soxhlet apparatus according to the method described in Bengana's work (Bengana et al., 2013). Approximately 10 g of lyophilized paste was used to extract oil with 150 mL petroleum ether at 40~60°C for 8–10 h. For each sample, three replicates were prepared and analyzed for each sample. We also used the olive oil for fatty acid composition analyses.

### Fatty acid composition analyses

We used GC-MS (Agilent 7000C, Agilent Technologies, USA) to analyze the fatty acid composition of the oil. Fatty acid methyl esters (FAMEs) were prepared by dissolving 0.1 g of oil in 2 mL of heptane and a solution of KOH (0.2 N) in methanol. Thereafter, the analyses were performed in accordance with a previously reported method (Bengana et al., 2013). GC-MS work condition: DB23 capillary column (30 m × 0.32 mm, 0.25 μm film thickness, Agilent Technologies, USA); the injection volume was 0.8 μL; linear velocity, 0.5 cm/min; split ratio of 1:30, v/v; interface, FID and the injector temperatures were 200, 280, and 250°C respectively. For each sample, three replicates were prepared and analyzed.

### Sugar content

The soluble sugars from 10 g of lyophilized fresh olive paste were extracted twice in 80% ethanol at 70°C and measured/ calculated by UNI 22608 method (Cecchi et al., 2013) using the G20S compact titrator (Thomas Scientific, USA). Concentrations were shown in g sugar/mL solution. The final sugar content was then referred to percentage of olive fruit. Three replicates were prepared and determined per sample.

### Phenolic and flavonoid content

A mixture of methanol and water (150 mL, 4:1 v/v) was added to 10 g of lyophilized paste, and the mixture was then shaken for 5 min with an Ultraturrax homogenizer. The solution was filtered using GF/F filter paper and hexane to remove the oil. The extract was concentrated to dryness using a Rotavapor at 40°C to a final volume of 10 mL. The quantity of phenolics and flavonoids in the extract were determined with the Folin-Ciocalteu procedure (Fernandez-Orozco et al., 2011) and the colorimetric assay (Brahmi et al., 2013) separately. The phenolic and flavonoid quantities were given as gram hydroxytyrosol equivalents per kilogram (HEQ) and as g luteolin equivalents per kg (LEQ) of fresh weight, respectively. And the standards of hydroxytyrosol, luteolin were purchased from Sigma-Aldrich (USA). Three replicates per sample were analyzed.

### Statistical analysis

The data were analyzed using Origin Pro 8 SR2 (Northampton, USA) and SPSS 22 (SPSS Inc., USA). SPSS 22 was used (SPSS Inc., USA) to perform an analysis of variances (ANOVA) with multiple A Posteriori mean comparisons. We used *p* < 0.05 to identify significant differences among all parameters analyzed. There was no significant difference between 2014's data and 2015's data (*p* < 0.05), so we didn't report it separately.

## Results

### Fruit growth and ripening

**Figure 1:**
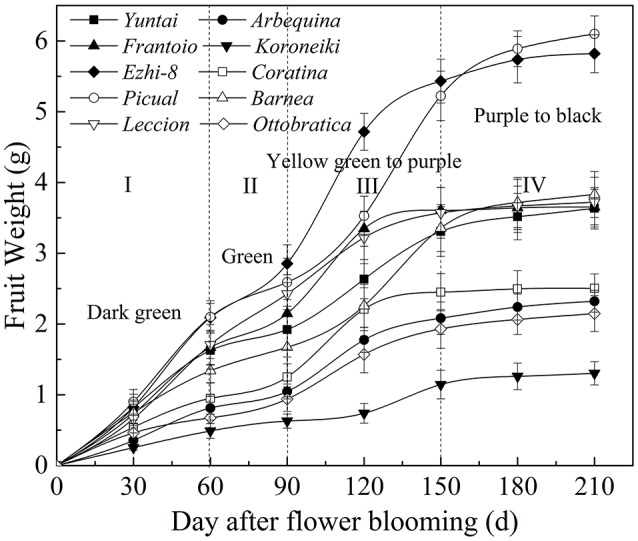
**Olive fruit weight and color change during growth and ripening**.

Olive fruits are drupes. The development stage of olives are divided into growth and ripening periods. In this study, the fruit weight and color change of olive fruits during growth and ripening are represented in Figure 1. The figure shows that the olive fruit growth and ripening processes were comparatively prolonged (greater than 210 d) and presented a special development trend: fast-slow-fast-slow, and the fruit color change was dark green-green-yellow green and purple-black. Therefore, the process was divided into four stages: phase I: 0–60 DAFB, phase II: 60–90 DAFB, phase III: 90–150 DAFB, and phase IV: 150–210 DAFB.

### The variation of qualitative characteristics during fruit development

**Figure 2:**
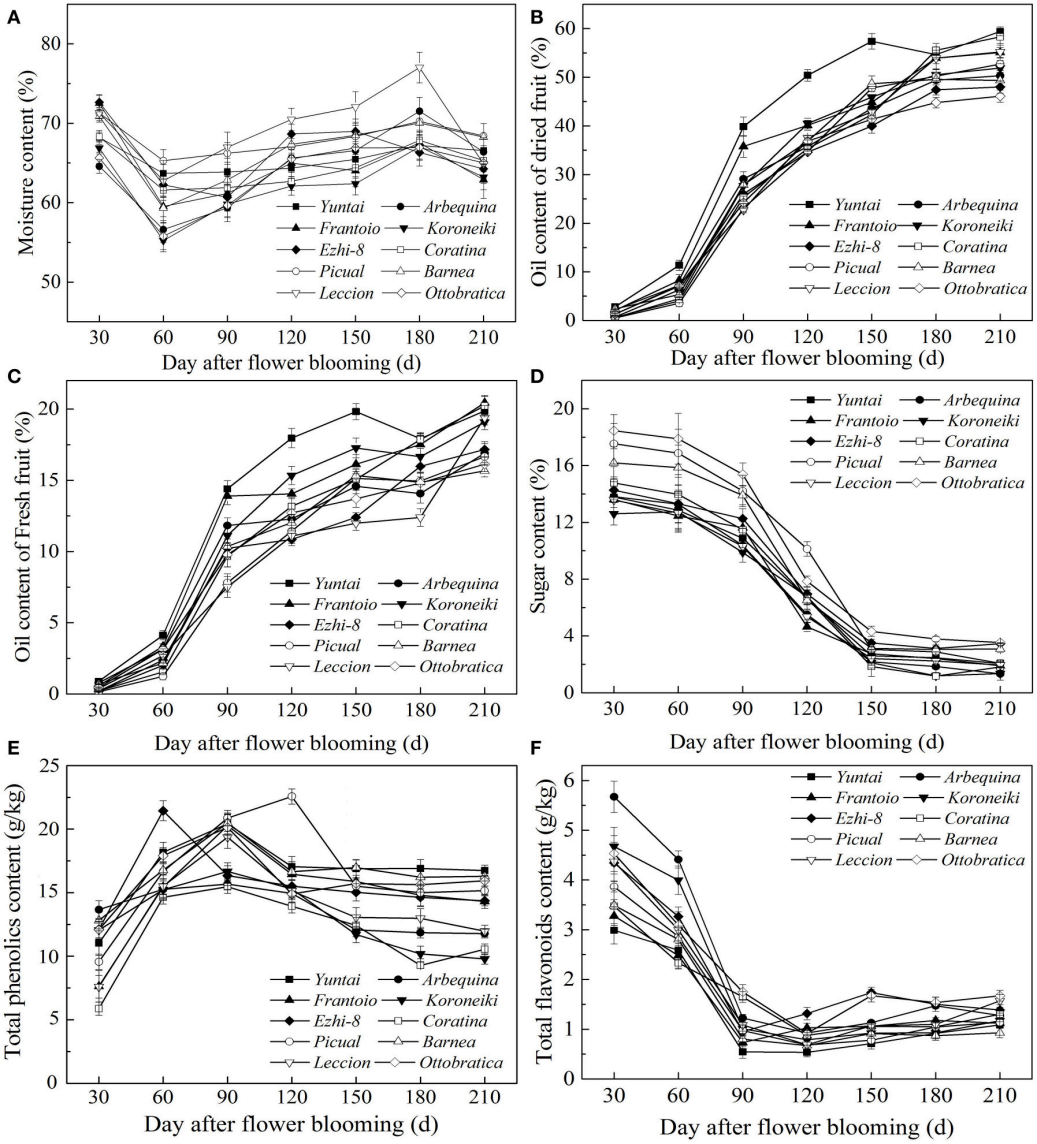
**Variation of qualitative characteristics during fruit development**. **(A)** Moisture content **(B)** Dried fruit oil content **(C)** Fresh fruit oil content **(D)** Sugar content **(E)** Total phenolics content **(F)** Total flavonoids content.

As olive fruit grows and ripens, biochemical processes occurred (Figure 2). During growth and ripening, A great fluctuation in moisture content were observed, and the content varied from 55.22 ± 0.81% to 77.01 ± 1.05% (Figure 2A). During 60–150 DAFB, the moisture content consistently increased and then decreased.

Oil accumulation of olive fruit during development is presented in Figures 2B,C. During fruit maturation, oil rapidly accumulated (Figure 2B). However, due to the unusual and rapid increase in the fruit moisture, the increase in fresh fruit oil content slowed down during 150–180 DAFB (Figure 2C). When fruits were fully ripened, the fresh and dried fruit oil content both reached a maximum, which ranged from 15.66 ± 0.51% to 20.44 ± 0.52% and from 46.12 ± 0.65% to 59.43 ± 0.63%, respectively. However, change in sugar content was opposite to oil accumulation (Figure 2D). The sugar content decreased rapidly as the fruit developing and reached a minimum at the end of the ripening period. In addition, the sugar content differed among the varieties during the growth period (*p* < 0.05); however, this difference began to decrease as the fruit ripened, which may be related to high oil accumulation during fruit ripening. (Figures 2C,D). These results also indicated that the high oil content is complementary for the low sugar content in olive fruit.

Throughout the development period, the phenolic content changed dramatically; the phenolic compound concentration varied from 22.48 ± 0.53 g/kg to 9.97 ± 0.35 g/kg (Figure 2E). In the early fruit growth stage (0–90 DAFB), the phenolics in the fruit were higher and increased gradually with the olive fruit development; thereafter, they began to decrease. During fruit ripening, the phenolic content was at a minimum. Flavonoids compose a class of phenolics, the evolution of which was not similar to other phenolics (Figure 2F). After the rapid decrease in 30–90 DAFB, the fruit flavonoid content slightly increased (90–210 DAFB).

### The qualitative characteristics of olive fruit at harvest

Generally, the olive harvested with good nutritional content showed well-balanced characteristics in quantity and quality indexes, such as fruit weight, oil content and phenolic content. Here, the major qualitative characteristics in each variety were determined at harvest time (Table 2). During harvesting, different olive variety fruits exhibited different contents of biochemical components. The moisture, dried fruit oil, fresh fruit oil, sugar, phenolic and flavonoid content of olive trees planted in southwest China ranged from 62.11 ± 0.64% to 68.39 ± 1.01%, 43.69 ± 1.39% to 56.02 ± 0.86%, 14.13 ± 0.50% to 20.44 ± 0.52%, 1.51 ± 0.33% to 4.06 ± 0.26%, 9.97 ± 0.35 g/kg to 16.90 ± 0.42 g/kg, and 0.81 ± 0.05 g/kg to 1.60 ± 0.11 g/kg, respectively. *Yuntai* exhibited the highest dried fruit oil content (56.02 ± 0.86%), highest phenolic content (16.90 ± 0.45 g/kg) and the lowest flavonoid content (0.81 ± 0.05 g/kg). *Frantoio* exhibited the highest fresh fruit oil content (20.44 ± 0.52%) and the highest flavonoid content (1.62 ± 0.08 g/kg). *Coratina* exhibited high fresh fruit and dried oil content (55.54 ± 1.20% and 20.23 ± 0.66%) but the lowest sugar and phenolic content (1.51 ± 0.33 g/kg and 9.97 ± 0.35 g/kg). Further, *Ezhi*-8 exhibited the lowest fresh fruit and dried oil content (43.69 ± 1.39% and 14.13 ± 0.50%) among the ten varieties.

**Table 2:** **The content of the biochemical components in olive fruit at harvesting**.

**Variety**	**Moisture content/%**	**Dried fruit oil content/%**	**Fresh fruit oil content/%**	**Sugar content/%**	**Phenolic content/(g/kg)**	**Flavonoid content/(g/kg)**
*Yuntai*	66.33 ± 0.76^bc^	56.02 ± 0.86^a^	18.86 ± 0.51^c^	2.53 ± 0.25^cd^	16.90 ± 0.45^b^	0.81 ± 0.05^e^
*Arbequina*	65.77 ± 0.76^c^	49.40 ± 1.84^bc^	16.91 ± 0.67^d^	2.01 ± 0.26^ef^	11.84 ± 0.33^de^	1.12 ± 0.12^c^
*Frantoio*	62.11 ± 0.64^a^	53.93 ± 1.43^ab^	20.44 ± 0.52^a^	2.58 ± 0.32^cd^	14.83 ± 0.30^de^	1.62 ± 0.08^a^
*Koroneiki*	62.07 ± 0.97^e^	50.33 ± 1.49^b^	19.09 ± 0.53^bc^	1.65 ± 0.30^fg^	10.26 ± 0.23^cd^	0.91 ± 0.06^de^
*Ezhi-8*	67.64 ± 0.59^ab^	43.69 ± 1.39^d^	14.13 ± 0.50^f^	3.32 ± 0.25^b^	14.37 ± 0.27^ef^	1.08 ± 0.09^c^
*Coratina*	63.58 ± 0.97^de^	55.54 ± 1.20^a^	20.23 ± 0.66^ab^	1.51 ± 0.33^fg^	9.97 ± 0.35^f^	1.30 ± 0.10^b^
*Picual*	66.85 ± 0.96^dc^	50.23 ± 1.43^b^	16.65 ± 0.60^de^	2.97 ± 0.26^bc^	14.44 ± 0.39^cd^	0.92 ± 0.06^de^
*Barnea*	68.39 ± 1.01^e^	49.57 ± 1.03^bc^	15.67 ± 0.44^e^	3.09 ± 0.13^b^	16.35 ± 0.23^cd^	1.05 ± 0.07^cd^
*Leccino*	64.03 ± 0.98^d^	53.93 ± 1.16^ab^	19.40 ± 0.50^abc^	2.32 ± 0.22^de^	12.89 ± 0.42^a^	0.89 ± 0.06^d^
*Ottobratica*	63.99 ± 0.79^d^	44.79 ± 1.07^cd^	16.13 ± 0.52^de^	4.06 ± 0.26^a^	15.67 ± 0.38^bc^	1.60 ± 0.11^a^

*The value of the biochemical components content in column with different letters are significantly different (p < 0.05), and the fruit loading (kg per plant) of each variety are separately: Yuntai: 15.5 ± 3.0, Arbequina: 22.0 ± 2.0, Frantoio: 16.3 ± 2.5, Koroneiki: 28.7 ± 3.5, Ezhi-8: 30.0 ± 1.8, Coratina: 25.1 ± 5.4, Picual: 15.1 ± 3.4, Barnea: 20.1 ± 2.4, Leccino: 10.1 ± 1.2, Ottobratica: 26.1 ± 2.4*.

*Yuntai* (20.44 ± 0.52%) exhibited higher fresh fruit oil content than *Coratina* (18.86 ± 0.51%) due to the difference in moisture content (Table 2). This suggests that moisture content is an important factor that determines the oil content in the olive varieties.

Olive oil fatty acid composition is another important characteristic for evaluating the olive varieties. The oil fatty acid composition of olive planted in China at harvesting (150–180 DAFB) was determined (Table 3). The oleic acid (C18:1, a monounsaturated fatty acid, MUFA) was the most abundant fatty acid (62.22–77.57%), and the linoleic (C18:2) was the most abundant poly unsaturated fatty acid (PUFA, 4.03–12.80%). Palmitic (C16:0) and stearic (C18:0) were the main saturated fatty acids (SFA, 12.25–22.38% and 2.11–4.88%, respectively). Therefore, olive oil is highly enriched in terms of unsaturated fatty acids (UFA), varying from 72.15 to 84.12%; MUFA, which was the main ingredients of the UFA, and consisted 64.59–78.51% of olive oil. Among the 10 varieties, *Yuntai* exhibited the highest UFA (84.12%) and highest MUFA content due to its high oleic content and low palmitic content, while *Arbequina* exhibited the lowest UFA content (75.15%). Due to the high linoleic levels (12.79 and 9.22%, respectively), the *Ezhi* and *Ottobratica* UFA levels were significantly greater than in *Arbequina* despite of low oleic levels (62.14 and 66.51%, respectively).

**Table 3:** **Fatty acid composition of olive oil at harvesting^a^**.

**Varieties**	**Percentage in oil/%**									
	**C16:0**	**C16:1**	**C18:0**	**C18:1**	**C18:2**	**C18:3**	**C20:0**	**C20:1**	**MUFA**	**UFA**
*Yuntai*	12.25 ± 0.92^f^	0.63 ± 0.10^ef^	3.35 ± 0.25^b^	77.57 ± 1.61^a^	4.37 ± 0.35^e^	1.24 ± 0.08^cd^	0.31 ± 0.09^de^	0.28 ± 0.08^cd^	78.51 ± 1.71^a^	84.12 ± 1.79^a^
*Arbequina*	22.38 ± 1.03^a^	2.46 ± 0.09^a^	2.16 ± 0.36^f^	63.77 ± 0.93^e^	6.57 ± 0.36^d^	1.93 ± 0.20^a^	0.42 ± 0.03^bc^	0.30 ± 0.07^bc^	66.65 ± 1.12^e^	75.15 ± 1.25^g^
*Frantoio*	17.39 ± 0.53^c^	1.26 ± 0.12^cd^	2.62 ± 0.24^e^	69.03 ± 1.01^c^	7.37 ± 0.24^c^	1.36 ± 0.17^c^	0.48 ± 0.05^ab^	0.49 ± 0.06^a^	70.77 ± 1.21^d^	79.50 ± 1.28^f^
*Koroneiki*	12.78 ± 0.39^f^	0.48 ± 0.05^f^	4.88 ± 0.69^a^	74.06 ± 1.32^b^	6.13 ± 0.56^e^	1.03 ± 0.12^def^	0.36 ± 0.07^cd^	0.29 ± 0.09^c^	74.89 ± 1.38^bc^	82.05 ± 1.46^bc^
*Ezhi-8*	19.09 ± 0.27^b^	2.12 ± 0.21^b^	2.11 ± 0.12^f^	62.22 ± 1.56^f^	12.80 ± 0.68^a^	1.26 ± 0.09^cd^	0.25 ± 0.09^e^	0.15 ± 0.08^e^	64.59 ± 1.61^f^	78.65 ± 1.59^f^
*Coratina*	15.92 ± 0.89^d^	0.33 ± 0.06^f^	3.06 ± 0.15^bcd^	74.50 ± 0.99^b^	4.34 ± 0.64^e^	1.24 ± 0.06^cd^	0.32 ± 0.10^de^	0.29 ± 0.5^c^	75.15 ± 1.02^bc^	80.73 ± 1.23^de^
*Picual*	15.36 ± 0.35^d^	1.03 ± 0.11^d^	3.25 ± 0.41^bc^	74.87 ± 1.78^b^	4.03 ± 0.28^e^	0.93 ± 0.14^ef^	0.31 ± 0.06^de^	0.21 ± 0.06^de^	76.22 ± 1.82^b^	81.17 ± 1.75^cd^
*Barnea*	14.63 ± 0.54^e^	1.03 ± 0.08^d^	2.76 ± 0.32^de^	74.91 ± 1.52^b^	5.32 ± 0.19^f^	0.82 ± 0.16^f^	0.32 ± 0.11^de^	0.21 ± 0.03^de^	76.26 ± 1.59^b^	82.40 ± 1.61^b^
*Leccino*	13.73 ± 0.23^e^	0.70 ± 0.02^e^	2.98 ± 0.38^cd^	74.37 ± 0.35^b^	6.45 ± 0.55^de^	1.12 ± 0.06^cde^	0.34 ± 0.10^cde^	0.31 ± 0.09^bc^	75.41 ± 0.45^b^	82.98 ± 0.78^b^
*Ottobratica*	17.10 ± 0.65^c^	1.55 ± 0.19^c^	2.59 ± 0.10^e^	66.96 ± 0.89^d^	9.28 ± 0.88^b^	1.63 ± 0.11^b^	0.52 ± 0.14^a^	0.37 ± 0.10^b^	69.03 ± 0.91^d^	79.93 ± 1.01^ef^

a *Saturated acid: C16:0, palmitic; C18:0, stearic; C20:0, eicosanoic; Unsaturated acid: C16:1, palmitoleic; C18:1, oleic; C18:2, linoleic; C18:3, linolenic; C20:1, eicosenoic. UFA, unsaturated fatty acid; MUFA, monounsaturated fatty acid*.

b *Means in a column with different letters are significantly different (p <0.05)*.

## Discussion

**Figure 3:**
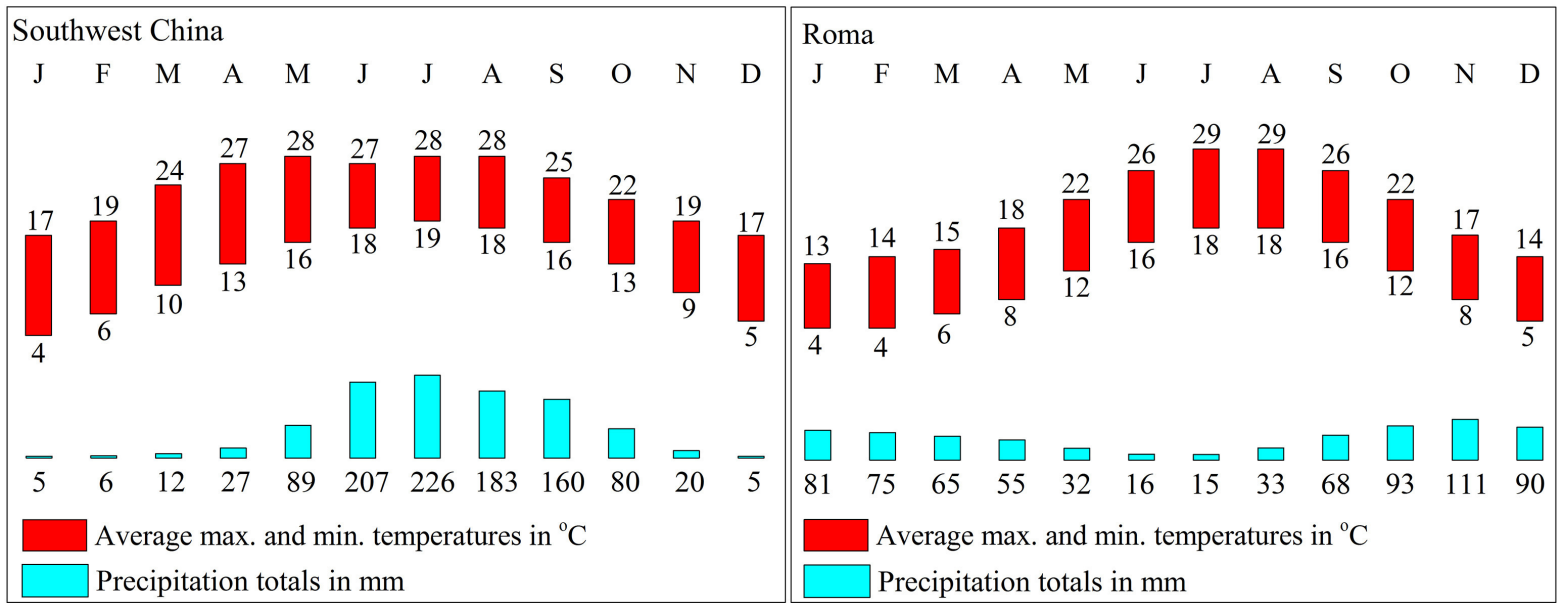
**Rainfall and temperature in southwest China and the Mediterranean region**. The data are from the China Meteorological Administration and World Meteorological Organization.

In China, olive trees are mainly planted in the southwest, which features the same latitude distribution as Mediterranean regions. However, the climate and environment are different. In southwest China, the weather is dry in winter and wet in summer and 70% of the rainfall fall during the summer, which is different from the Mediterranean region (Figure 3). Furthermore, southwest China experiences approximately 300–500 mm more precipitation and the average altitude of planting is 500 m higher than the ones in the Mediterranean region.

### The qualitative characteristics of olive planted in china

**Figure 4:**
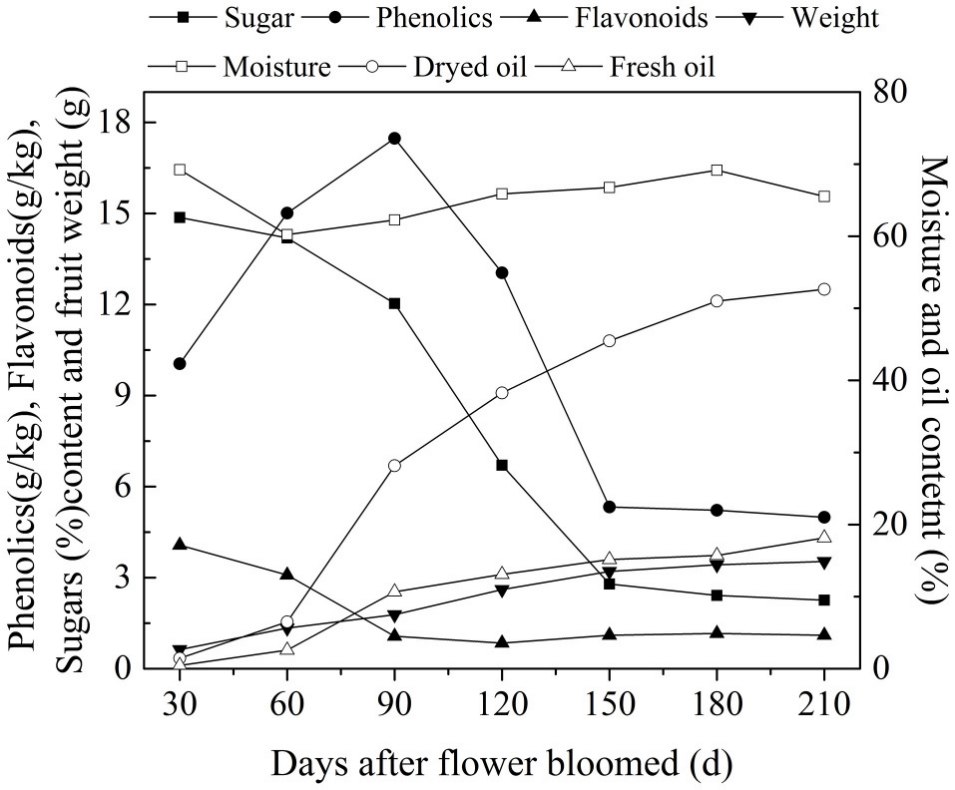
**The variation of biochemicals content in olive fruit of southwest China**. The data are the average value of the 10 varieties.

**Figure 5:**
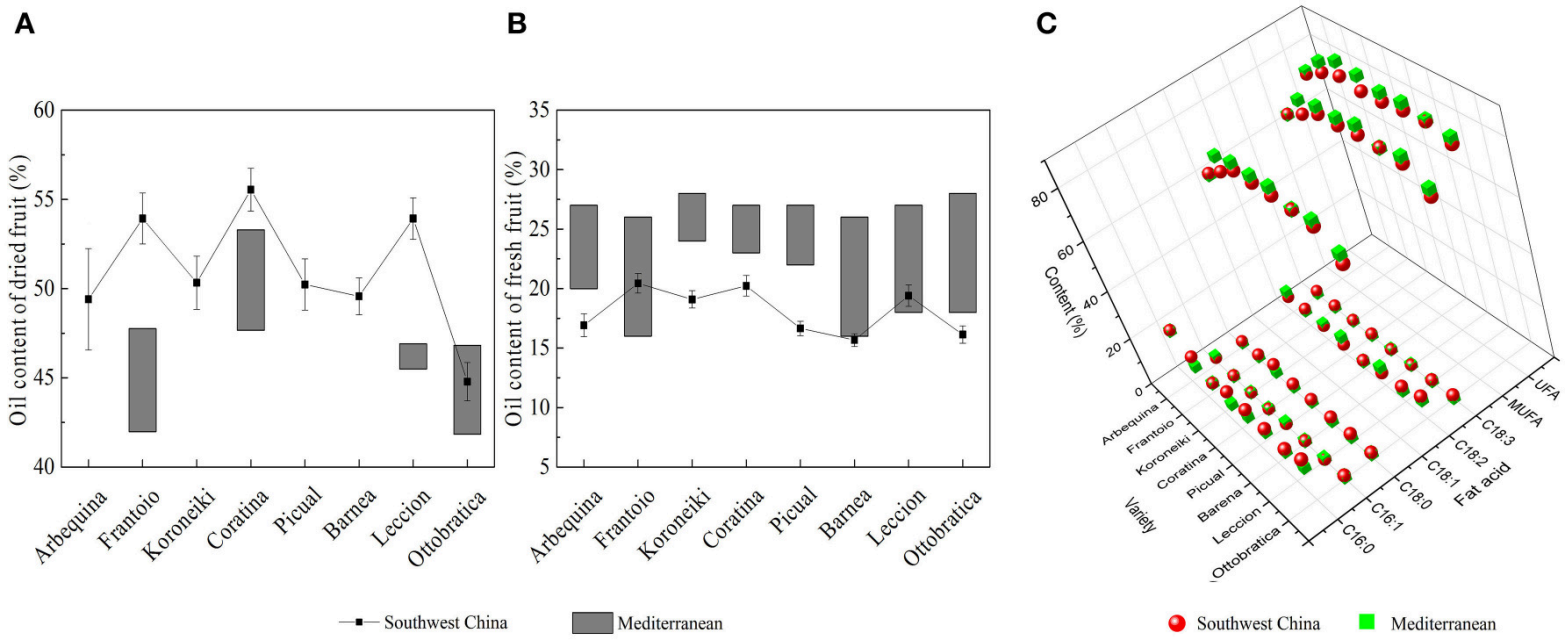
**The oil content and fatty acids composition of olives from southwest China and the Mediterranean region**. **(A)** Dried fruit oil content **(B)** Fresh fruit oil content **(C)** Fatty acids composition of olive oil. The data of Mediterranean olive are from the olive database.

After decades of cultivation in southwest China, the biochemical content in olive during fruit development (Figure 4) is consistent with Mediterranean-grown olives (Conde et al., 2008). However, during fruit development fruit weight increased and sugar content decreased rapidly. The rapid increase in fruit weight can be attributed to heavy rainfall during summer and autumn months while in Mediterranean regions it is the dry season. (Figure 3). This indicates that olive trees grown in high moisture will produce fruits with high moisture content. This result agrees with the findings that sufficient irrigation in fruiting stage will raise fruit water content (Proietti and Antognozzi, 1996). In addition, after decades of cultivation, oil content of olive planted in Southwest China has changed only slightly compared with data from *Olea* Databases^1^. The dried fruit oil contents of *Frantoio, Coratina*, and *Leccino* increase by about 5% (Figure 5A), while the fresh fruit oil contents of these varieties have decrease due to high moisture content (Figure 5B). Therefore, moisture content also contribute to the difference in the oil contents between olives from Southwest China and the Mediterranean region.

Moreover, olive planted in China show a different fatty acid composition. The linoleic acid (C18: 3) content of Chinese olive is ranged from 0.82 to 1.92%, which is much higher than data that provided by International Olive Council^2^ (<1%) (2008). Also, due to the higher palmitic (C16:0) and stearic (C18:0) content as well as lower oleic (C18:1) content, the UFA and MUFA content in olive fruits from Southwest China was significantly lower than in the fruits from the Mediterranean region (Figure 5C). In Nergiz and Ergönül (2009) and Ouni et al. (2011) research, it has been discovered that olives grown at a high altitude tend to exhibit high oleic and UFA content. Therefore, the high altitude of Southwest China may cause the low UFA content. Furthermore, Temime et al. (2006) reports that olive fruits from cooler areas exhibit more unsaturated fatty acid than fruits from dry and warm areas. Southwest China is warmer than the Mediterranean region (Figure 3), which may also play a role in the lower UFA and MUFA yields in Southwest China olive fruit. These results are in agreement with the findings of other researchers that plants with high unsaturated fatty acid content usually show strong cold-resistant (da Cruz and Bazana, 2010). Inconsistent with (Molina et al., 2012) report, *Arbequina* had a poor oil stability quality due to low MUFA content (66.65 ± 1.12%) which resulted from the high palmitic acid (C16: 0) content (22.38 ± 1.03%).

Phenolic compounds, which include phenolic acids, phenolic alcohols and flavonoids, are one of the most important minor components and directly affect the fruit quality (Fernandez-Orozco et al., 2011; Brahmi et al., 2013). In this study, olive manifest a similar phenolic content and has a wide range of phenolics content between olive varieties (Table 2), such as the Coratina and Koroneiki have lower Phenolic content (<10 g/kg), while Frantoio and Barnea have higher Phenolic content (>14 g/kg) (Vinha et al., 2005; Cecchi et al., 2013). However, the change in phenolic content during fruit development was more rapid than in the Mediterranean region (Vossen, 1998). In Southwest China, phenolic content in olive fruit reaches a maximum from July to August (90–120 DAFB), but olive fruit in the Mediterranean region reaches a maximum from October to November (Figure 2E). All these differences in olive between China and Mediterranean offer a manifestation that the olive has adapted to the southwest China environment and climate.

### The relationship between oil and sugar content

**Figure 6:**
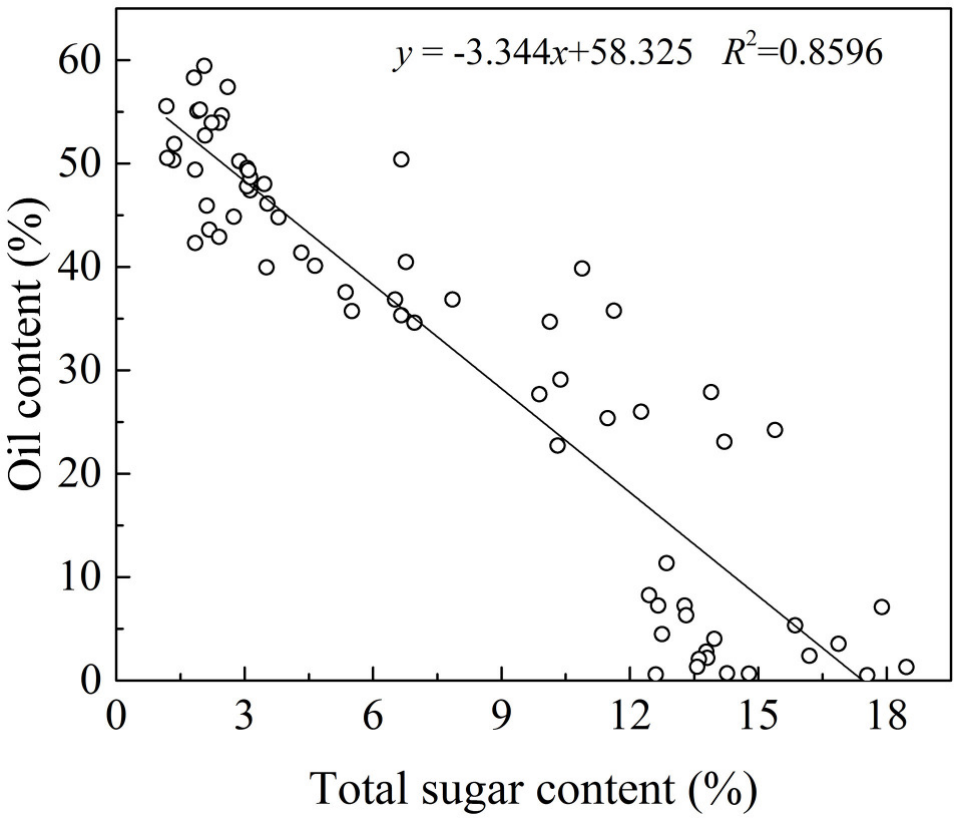
**The correlation between oil and sugar content**.

In plant cells, sugar is required for lipids biosynthesis. Acetyl-CoA is the initial substrate for fatty acids synthesis, and the synthesis of Acetyl-CoA requires pyruvate produced from sugar via glycolysis (Sánchez and Harwood, 2002). In olive plants, there are two sources of sugars for lipid biosynthesis (Proietti et al., 1999). One is from mature leaves and the other is through photosynthesis in the fruits themselves (Conde et al., 2008). Although the sugar from mature leaves is considered as the major source, some researches have showed that photosynthesis in the olive fruits also play an important role in fruit growth and lipids biosynthesis (Sánchez, 1995). In fact, oil in the olive fruit accumulated while the sugars decease rapidly (Figure 2). This negative correlation between the oil and sugar content (*p* < 0.01, Figure 6 is consistent with results for olive trees planted in the Mediterranean region (Cherubini et al., 2009; Migliorini et al., 2011), and suggests that sugars are the source for the biochemical oil accumulation process during olive development and ripening and vice versa.

## Conclusion

After several decades of cultivation and domestication in Southwest china, olives have adapted to the local environment and the differences are expressed in its unique qualitative characteristics. More precipitation during fruit development makes the olives in China have a higher moisture content. This leads to lower oil content and unsaturated fatty acid percentages in fresh fruits. Furthermore, lowered sugar content in the olive fruit is the direct result of large scale biosynthesis of fatty acids.

## Author contributions

ZC and MZ conducted the experiments. ZC, HC, ZY, and QH designed the experiments. KZ and QH revised the manuscript. ZC analyzed the data and drafted the manuscript.

## Funding

This work was funded by Research Fellowships from the Sichuan Science and Technology Department (12ZC2220), and supported by Sichuan Liangshan New Technology Development Co.,Ltd.

### Conflict of interest statement

The authors declare that the research was conducted in the absence of any commercial or financial relationships that could be construed as a potential conflict of interest. The reviewer SW declared a shared affiliation, though no other collaboration, with one of the authors KZ to the handling Editor, who ensured that the process nevertheless met the standards of a fair and objective review.

## Footnotes

^1^Oleadb. *Olea* Databases. Available online at: http://www.oleadb.it/olivodb.html.

^2^http://www.internationaloliveoil.org.
